# A Crown Ether-Based Covalent Organic Polymer Composite Membrane and Its Application in Molecular Separation

**DOI:** 10.3390/membranes16020056

**Published:** 2026-02-02

**Authors:** Yike Chen, Wenju Shi, Meitong Liu, Zhihong Huang, Jianshe Hu, Zhangpei Chen

**Affiliations:** Center for Molecular Science and Engineering, College of Science, Northeastern University, Shenyang 110819, China; 20232037@stu.neu.edu.cn (Y.C.); 20212239@stu.neu.edu.cn (W.S.); 2470055@stu.neu.edu.cn (M.L.); 20222231@stu.neu.edu.cn (Z.H.); hujs@mail.neu.edu.cn (J.H.)

**Keywords:** covalent organic polymer, crown ether, nanofiltration membrane, membrane separation technology, dye desalination

## Abstract

Organic dyes are critical components in industries ranging from textiles, plastics, and paper to food, cosmetics, and pharmaceuticals. However, their widespread use leads to significant environmental pollution. Consequently, developing efficient methods to treat dye wastewater is urgently needed. In this work, a high-performance composite membrane was developed with a poly(dibenzo-18-crown-6) covalent organic polymer (COP) interlayer. The chemical structure of the COP was verified by FT-IR, and BET analysis indicated that the as-synthesized material possesses a predominantly mesoporous structure with a minor microporous contribution. Subsequently, the membrane was fabricated by depositing a COP colloid on a nylon-66 support via vacuum filtration, followed by the formation of a dense polyamide (PA) active layer through interfacial polymerization (IP) between amine and acyl chloride monomers. Systematic evaluation of dye separation performance using a cross-flow filtration setup identified optimal operating conditions. Under these conditions, the membrane demonstrated effective molecular sieving behavior, achieving both high dye rejection and favorable solvent permeability. In long-term stability tests, the membrane maintained a rejection rate of over 99% for Congo red over 48 h, while sustaining a water flux of 103.2 L m^−2^ h^−1^ bar^−1^ (LMH/bar). Furthermore, the membrane exhibited promising potential for dye desalination applications, achieving a high Congo red/potassium chloride separation selectivity of 186.8 with a flux of 138.2 LMH/bar. This study confirms that the poly(dibenzo-18-crown-6)-based composite membrane is a reliable and efficient material for molecular separation in wastewater treatment.

## 1. Introduction

Global efforts toward sustainable development, including commitments to energy conservation and emission reduction, demand urgent action to solve the severe water contamination issues [1]. Coupled with escalating freshwater scarcity, this challenge renders the advancement of efficient and ecologically benign wastewater purification technologies a critical scientific and engineering priority [2]. Specifically, it is imperative to solve the problem of industrial organic dye pollution, as these persistent pollutants deteriorate aquatic environments and pose significant bioaccumulation risks [3]. Given this context, membrane separation technology has become a focus for desalination and dye-wastewater remediation, owing to its advantages of operational simplicity, relatively low energy consumption, and favorable economic prospects [4,5]. Among membrane separation technologies, nanofiltration (NF) has gained prominence for treating dye-wastewater, specifically due to its high efficiency in removing dyes and salts while operating at lower pressures than reverse osmosis, offering a balanced and sustainable solution [6].

Conventional nanofiltration (NF) membranes, predominantly fabricated from materials such as polyamide (PA) [7], sulfonated polyethersulfone (SPES) [8], and polyelectrolytes [9], operate on a separation mechanism involving both size exclusion and electrostatic repulsion (the Donnan effect) [10,11,12]. However, the broader application of these polymeric membranes is constrained by two intrinsic limitations [13]. Firstly, they are subject to a fundamental trade-off between permeability and selectivity, where enhancements in one property typically compromise the other, imposing a critical bottleneck on separation efficiency and application potential [14,15,16,17]. Secondly, the amorphous, disordered microstructure of conventional polymers presents a significant barrier to precise control over surface chemistry and pore size distribution [18]. The emergence of mixed-matrix membranes (MMMs) provides a feasible solution to simplify the trade-off effect by introducing a discrete phase of micro- or nano-sized particles as the fillers or interlayer to the thin-film composite (TFC) structure [19,20,21,22,23,24]. Recently, covalent organic polymers (COPs) have emerged as a highly promising platform for nanofiltration, demonstrating significant potential in applications such as dye wastewater treatment and desalination [25]. The exceptional interest in COPs is driven by their unique structural advantages [26]. Their modular nature permits the precise, molecular-level design of building blocks and pore systems, while their highly ordered frameworks provide abundant sites for post-synthetic modification with tailored functional groups [27]. Combined with notable chemical stability, these features position COPs as versatile candidates for advanced separation processes [28]. However, translating this material-level promise into practical, high-performance membranes presents substantial challenges [29].

**Figure 1:**
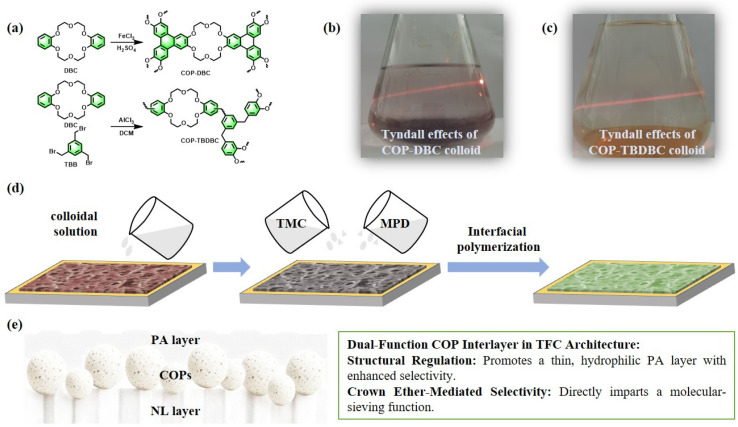
(**a**–**d**) Schematic illustration for the preparation of the COPs (COP-DBC and COP-TBDBC) and the corresponding MMMs (CM-DBC and CM-TBDBC). (**e**) A conceptual transport model of dual-function COP interlayer in the TFC architecture.

In this study, a crown ether-based covalent organic polymer composite membrane was synthesized via a combination of ultrasonic dispersion and in situ interfacial polymerization (IP) processes. The strategy involved constructing an interlayer of two mesoporous COPs derived from benzocrown ethers within a thin-film composite (TFC) structure [30]. Specifically, an insoluble COP (denoted as COP-DBC) was first synthesized via FeCl_3_-catalyzed oxidative coupling of dibenzo-18-crown-6 [31]. For comparison, a second COP (termed COP-TBDBC) was prepared through Friedel–Crafts alkylation between dibenzo-18-crown-6 and 1,3,5-tris(bromomethyl)benzene [32]. Subsequently, these polymers were employed to engineer a composite membrane with a sandwich-like architecture. To construct a high-performance composite membrane, the synthesized COPs were initially dispersed into colloidal solutions via ultrasonication. The resulting colloids were then deposited as a conformal interlayer on a nylon-66 microfiltration support using vacuum filtration. Finally, interfacial polymerization (IP) between amine (m-phenylenediamine) and acyl chloride monomers was conducted over this interlayer to form a dense PA film [33]. The resultant three-layer sandwich-like composite architecture led to a substantial improvement in dye molecule interception. The impact of interlayer type and loading on membrane morphology and separation performance was systematically evaluated. Notably, the COP interlayer is designed to function as a dual-purpose component, acting both as a structural template that promotes the formation of a thinner, more hydrophilic PA layer with improved permeability–selectivity, and as a selective molecular sieve enabled by its crown ether motifs, as illustrated in Figure 1. The rejection performance of the membranes was tested against dyes, dye mixtures, and various salts, as well as long-term operational stability under continuous nanofiltration conditions. This research not only introduces an innovative membrane design strategy but also significantly expands the utility of COP materials in high-performance nanofiltration, thereby contributing to the development of highly permeable and economically viable membranes.

## 2. Materials and Methods

### 2.1. Materials and Characterizations

All chemical reagents were employed as received, without any further purification. Sourced from Aladdin Biochemical Technology Co., Ltd. (Shanghai, China) were the following: dibenzo-18-crown-6-ether (DBC), anhydrous FeCl_3_, AlCl_3_, 1,3,5-tris(bromomethyl)benzene, trimesoyl chloride (TMC), m-phenylenediamine (MPD), acetic acid, n-hexane, 1,4-dioxane, mesitylene, dichloromethane (DCM), and methanol. Sinopharm Chemical Reagent (Shanghai, China) supplied rhodamine B (RhB), acid fuchsin (AF), methylene blue (MB), Eriochrome Black T (EBT), methyl orange (MO), alcian blue 8GX (AB), 2-amino-5-nitrophenol (2-ANP), Congo red (CR), and ethylenediaminetetraacetic acid disodium salt (EDTA). The porous substrate, a Nylon-66 microporous membrane with a 0.22 μm pore size, was acquired from Tianjin Branch Billion Lung Experimental Equipment Co., Ltd. (Tianjin, China).

The morphology of all samples was determined with scanning electron microscopy (SEM, JEOL 6500F). The absorption wavelength was measured using a UV-vis spectrophotometer (TU-1901, Beijing Purkinje General Instrument Co., Ltd., Beijing, China). Other instruments are listed as follows: thermogravimetric analysis (TGA) was performed with a Diamond instrument (PerkinElmer Instruments, Shanghai, China), Fourier transform infrared (FT-IR) spectra were measured with samples pressed into KBr pellets on a PerkinElmer Spectrum One (B) spectrometer (PerkinElmer Instruments, Shanghai, China). N_2_ sorption at 77 K (BSD INSTRUMENT, BSD-660M) followed 12 h outgassing at 150 °C; BET areas and BJH pore sizes were calculated.

### 2.2. Synthesis of COPs

A 30 mL dichloromethane solution containing 4.7 g of anhydrous FeCl_3_ was prepared in a round-bottom flask under a nitrogen environment. To this mixture, 0.2 mL of H_2_SO_4_ was introduced. Separately, 1.5 g of DBC was dissolved in 25 mL of dichloromethane within a pressure-equalizing funnel. This monomer solution was then introduced into the FeCl_3_ suspension via dropwise addition over a 30 min period. The resulting mixture was stirred overnight, forming a dark greenish/blackish precipitate. The reaction was quenched with 15 mL of methanol, followed by 30 min of stirring. After filtration, the solid residue was sequentially washed with dichloromethane and methanol. To guarantee the thorough elimination of FeCl_3_, the material was subsequently rinsed with an EDTA solution and then water. The resulting purple solid was purified via Soxhlet extraction using methanol and subsequently dried, affording the final product COP-DBC as a purple-tinted solid powder.

Aluminum trichloride (3.75 mmol) was added to a DCM (20 mL) containing 1,3,5-tris(bromomethyl)benzene (0.5 mmol). The reaction mixture was stirred at 80 °C for 24 h. The resulting precipitate was sequentially washed with dilute hydrochloric acid (3 × 40 mL), methanol (20 mL), and acetone (20 mL). Subsequently, the obtained solid was continuously washed with methanol using a Soxhlet extractor for 24 h. The resulting solid was finally dried under reduced pressure at 60 °C for 24 h to yield a brown powdery product identified as COP-TBDBC.

### 2.3. Fabrication of the COP-Based Composite Membrane

The fabrication process for the TFN membranes is illustrated in Figure 1. A colloidal suspension of COPs was prepared by adapting a literature method [30]. In a standard procedure, a specific mass of the as-synthesized COPs powder was dispersed in absolute ethanol to achieve a concentration of 0.1 mg/mL; this mixture was then sonicated for 5 h to form a homogeneous colloid. A defined volume of this colloidal solution was vacuum-filtered onto a nylon-66 microporous membrane (NL, effective surface area: 12.56 cm^2^), which was subsequently transferred to a glass plate. An aqueous solution containing MPD was poured over the substrate and allowed to contact the surface for a few seconds. The excess diamine solution was cleared using a squeegee roller, and the surface was briefly air-dried. Subsequently, an n-hexane solution of TMC was applied to initiate the interfacial polymerization reaction for a few seconds, after which the organic solution was drained to halt the process. This yielded the final composite membrane incorporating the COPs interlayer. These membranes are referred to as CM-DBC and CM-TBDBC.

### 2.4. Dye Adsorption Tests

To evaluate the adsorption performance, the membrane was immersed in 30 mL of a 50 μmol/L dye solution. The residual dye concentration in the solution was determined at regular intervals using UV-Vis spectroscopy.

### 2.5. Filtration Performance Evaluation

The water flux of the membrane was evaluated by employing a custom-built cross-flow filtration setup [34]. The permeation performance, expressed as *P* (LMH/bar), was calculated using the following equation:

(1) $$P=\frac{V}{A\cdot t\cdot \Delta p}$$

Here, *V* (L) represents the volume of water passing through the membrane, *A* (m^2^) is the effective filtration area, *t* (h) is the duration of the experiment, and *ΔP* (bar) is the applied transmembrane pressure.

Separation studies were performed with feed solutions containing 50 μmol/L dyes or 1 g/L inorganic salts at an applied pressure of 0.1 bar. After a 20 min stabilization period at the operating pressure, permeate was collected over a 40 min interval. The concentrations of dyes in the feed and permeate were determined by UV–vis spectrophotometry, while salt concentrations were measured with a conductivity meter. The solute rejection (*R*) and dye/salt selectivity (*α*) were calculated using the following definitions:

(2) $$R=\left(1-\frac{C_{f}}{C_{P}}\right)\times 100\%$$

(3) $$\alpha =\frac{100-R_{salt}}{100-R_{dye}}$$

where *C_p_* and *C_f_* denote the solute concentrations in the permeate and feed solutions, respectively, while *R_salt_* and *R_dye_* signify the rejection values for salt and dye molecules, respectively.

The antifouling test was performed at 0.3 bar using a CR dye solution. Initially, the pure water flux (*J_w_*) was recorded every 20 min over the first hour, followed by measurement of the permeate flux (*J_c_*) during 5 h of filtration with the CR solution as the feed. After cleaning and soaking the membrane in deionized water for 2 h, the pure water flux was measured again under identical conditions (*J_w2_*). The antifouling performance was evaluated using the flux recovery ratio (FRR), which indicates the membrane’s ability to regain its initial flux after cleaning, and the flux decline ratio (FDR), which reflects the extent of flux loss during fouling. These metrics were calculated as follows:

(4) $$FRR(\%)=\;\frac{J_{w2}}{J_{w}}\times 100\%$$

(5) $$FDR(\%)=\left(1-\frac{J_{c}}{J_{w}}\right)\times 100\%$$

A high FRR suggests effective cleaning and reversible fouling, while a high FDR indicates significant flux loss during filtration, typically associated with more severe fouling.

## 3. Results and Discussion

### 3.1. Membrane Characterization

The fabrication of the composite membrane commenced with the synthesis of two distinct COPs. As depicted in Figure 1a, the insoluble polymer COP-DBC was synthesized via FeCl_3_-catalyzed oxidative coupling of dibenzo-18-crown-6. Accordingly, the second COP, termed COP-TBDBC, was prepared via Friedel–Crafts alkylation between dibenzo-18-crown-6 and 1,3,5-tris(bromomethyl)benzene. To fabricate COP-based interlayers, colloidal solutions (0.1 mg/mL) of COP-DBC and COP-TBDBC were first prepared by ultrasonic dispersion, evidenced by a clear Tyndall effect (Figure 1b,c). Using vacuum filtration, the colloids were first deposited as a conformal interlayer on a nylon-66 support. A dense PA selective layer was then formed in situ via interfacial polymerization (IP) between MPD and TMC, constructing the final three-layer, sandwich-like composite membrane. Subsequently, the structure and morphology of both the resultant membrane and its precursor materials were characterized using a comprehensive suite of techniques.

**Figure 2:**
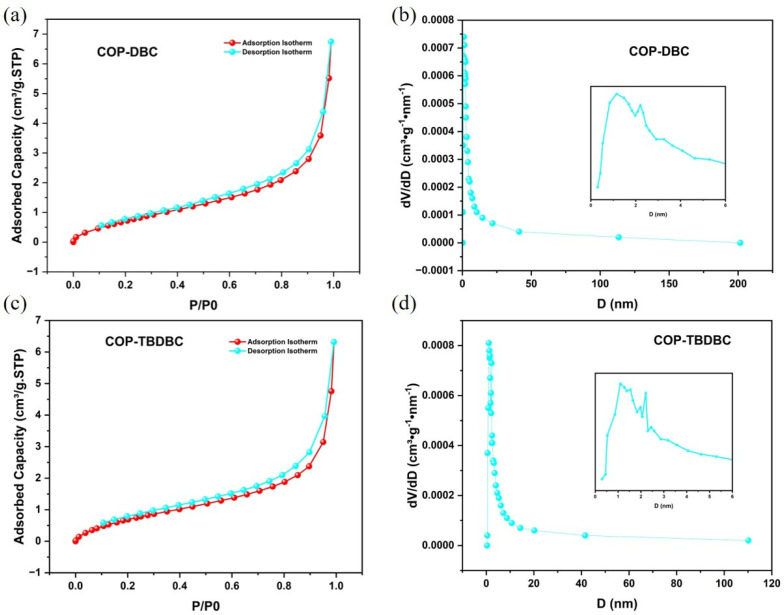
N_2_ adsorption and desorption isotherm and pore size distribution analyses of COP-DBC (**a**,**b**) and COP-TBDBC (**c**,**d**).

Firstly, the porosity of the synthesized COP-DBC and COP-TBDBC was systematically investigated (Figure 2). Nitrogen physisorption measurements at 77 K revealed that the prepared COPs exhibited type II isotherms, with hysteresis loops in both medium- and high-pressure regions, indicating a mostly mesoporous structure with a small portion of microporous structure. For COP-DBC, the multipoint BET surface area (measured at *P*/*P*_0_ = 0.0954–0.3082) was 3.3487 m^2^/g, with a Langmuir surface area of 6.31 m^2^/g. COP-TBDBC exhibited slightly lower values of 3.1456 m^2^/g (BET) and 6.1218 m^2^/g (Langmuir). Pore size distribution analysis using nonlocal density functional theory (NLDFT) indicated a combined micro- and meso-porous structure for both materials. BJH analysis of the adsorption data yielded average pore diameters (4 V/A method) of 8.7989 nm for COP-DBC and 9.0590 nm for COP-TBDBC. Notably, the most probable pore diameters from adsorption were very close for both samples, each measuring around 2.08 nm.

**Figure 3:**
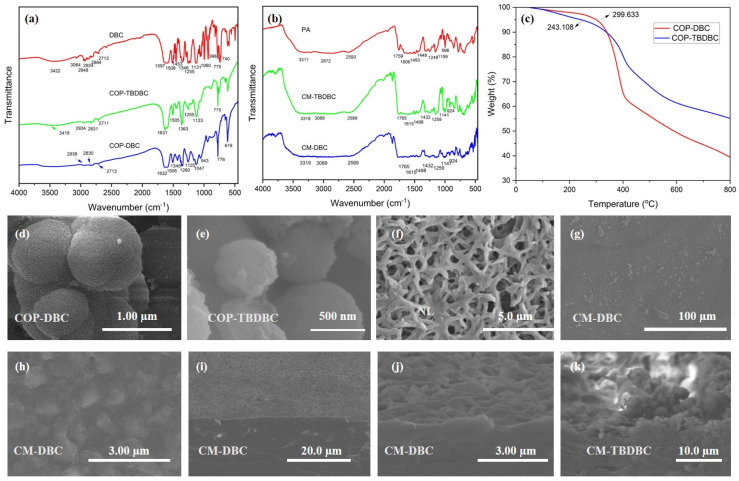
FT-IR analyses of COPs (**a**) and MMMs (**b**). TGA analyses of COP-DBC and COP-TBDBC (**c**). SEM images of COP-DBC (**d**) and COP-TBDBC (**e**). SEM image of nylon-66 membrane (**f**). SEM images (**g**,**h**) and cross-sectional morphology of MMMs (**i**–**k**).

The FT-IR spectra of DBC, COP-DBC, and COP-TBDBC are presented in Figure 3a. The infrared spectrum of DBC confirmed the presence of its key functional groups. Stretching vibrations were identified for aromatic C–H (3064 cm^−1^) and aliphatic C–H (2948–2884 cm^−1^). The phenyl groups contributed to in-plane ring vibrations at 1597 and 1506 cm^−1^. The vibrational signatures of the crown ether unit were evident from the strong ν(Ph–O–C) stretching bands at 1255 and 1131 cm^−1^. The absorption band observed at 3422 cm^−1^ can be attributed to the O–H stretching vibration of water molecules trapped within the cavity of the crown ether. For COP-DBC, the disappearance of characteristic aromatic C–H peaks, along with the emergence of prominent aromatic skeleton vibrations (e.g., C=C stretches at 1632 cm^−1^ and 1260 cm^−1^), indicates the formation of new C–C bonds via FeCl_3_-catalyzed oxidative coupling. These spectral features, particularly the band at 1260 cm^−1^, which is characteristic of olefinic C=C stretching in polycyclic aromatics, are consistent with the formation of a triphenylene structure, as supported by the literature [31]. FT-IR analysis of COP-TBDBC revealed alkyl C–H stretching (2934–2831 cm^−1^), and a strong C–H bending vibration (1363 cm^−1^). These features provide clear evidence for the extensive incorporation of TBB units into the crown ether structure. Moreover, the FT-IR analysis of the fabricated MMMs was also carried out, and the results are provided in Figure 3b. Analysis of a separately prepared pure PA layer revealed strong and well-defined characteristic infrared absorptions, an N–H stretch at 3311 cm^−1^, an amide C=O stretch at 1759 cm^−1^, and an aromatic skeletal stretch at 1606 cm^−1^. However, the infrared signals from the interlayer COPs were obscured because of the dominant absorption from the covering PA layer. Next, the TGA was performed by heating several milligrams of the sample in an alumina crucible from 50 °C to 800 °C at 10 °C/min under a nitrogen flow. The resulting thermograms (Figure 3b) indicated onset decomposition temperatures of 299.6 °C and 243.1 °C, respectively, confirming the good thermal stability of both materials. COP-DBC exhibited a sharp 37 wt% weight loss in the range of 331 °C to 410 °C, which is likely due to the decomposition of its ether groups. By contrast, COP-TBDBC decomposed more gradually. We attribute this decomposition profile to its structural motif; the increased benzene ring content imparts enhanced rigidity and likely mitigates the thermal vulnerability of the crown ether units through a combination of steric shielding and electronic stabilization.

**Figure A1:**
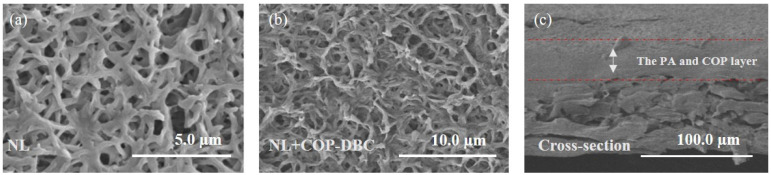
SEM images of NL (**a**) and NL+COP-DBC (**b**). Cross-sectional morphology of CM-DBC (**c**).

Then, the SEM images of the COPs and the membrane surface, cross-section morphology of the MMMs were characterized (Figure 3). In the case of COP-DBC, the observed morphology was an accumulation of agglomerated microspheres with moderately polydisperse sizes, averaging around 550 nm. A comparable spherical morphology was observed for COP-TBDBC, but with a reduced average sphere diameter of around 500 nm. The pristine nylon membrane exhibited a porous surface with a crossed and interlaced fiber structure (Figure 3g). The sequential deposition of the COP interlayer and PA layer completely covered the fibrous nylon support, eliminating pinholes and generating a distinctive “bulge–plateau” surface morphology. A layered architecture was further confirmed by cross-sectional SEM (Figure 3 and Figure A1), as visualized in the composite membranes CM-DBC and CM-TBDBC: a dense PA top layer overlying a distinct COP interlayer. These results confirm the successful encapsulation of COPs within the PA matrix to form integrated composite membranes.

### 3.2. Molecular Sieving Performance of the Membranes

The influence of COP-DBC loading on membrane performance was systematically investigated to optimize both the fabrication process and separation efficiency of the resulting composite membrane. As summarized in Table 1, water permeance declined progressively with increasing COP-DBC loading. For Congo red (CR), permeance decreased from 254.2 to 132.6 LMH/bar, and for Acid Fuchsin (AF), it fell from 580.3 to 193.4 LMH/bar. Conversely, dye rejection improved with higher COP-DBC content. AF rejection rose from 58.2% to 89.3%, while CR rejection, already elevated due to its larger molecular size, increased only modestly from 97.6% to 99.9%. The observed decline in permeance is primarily attributed to increased hydraulic resistance as the COP-DBC layer becomes denser at higher loadings. Furthermore, the molecular size of CR exceeds the intrinsic nanopore dimensions of COP-DBC, leading to surface enrichment and additional transport resistance. Although a loading of 2.5 mg delivered the highest overall separation performance, a loading of 2.0 mg was selected for subsequent studies to better balance permeance with rejection.

The separation performance of COP-DBC-based composite membranes was systematically optimized by investigating the effects of key IP parameters: aqueous MPD concentration, TMC concentration in n-hexane, and IP reaction time. The results are summarized in Table 2. Increasing the MPD concentration from 2.0 to 5.0 wt% led to a significant rise in water permeance, accompanied by a gradual decline in dye rejection. For AF, permeance increased from 127.1 to 690.8 LMH/bar, while rejection decreased from 98.4% to 64.2%. This trend suggests that higher MPD concentrations promote the formation of a more open pore structure in the PA layer, enhancing water permeance at the expense of size selectivity. Based on the balance between permeance and rejection, 3.5 wt% MPD was selected for subsequent studies. The influence of TMC concentration exhibited a non-monotonic behavior. As TMC concentration increased from 0.15 to 0.25 wt%, AF permeance rose from 138.2 to 381.3 LMH/bar, with rejection decreasing from 95.5% to 87.3%. Further increasing TMC to 0.35 wt% caused permeance to drop to 165.8 LMH/bar while rejection recovered to 92.3%. These variations are attributed to changes in the MPD/TMC stoichiometric ratio, which modulates the cross-linking density and thickness of the PA layer. An optimal TMC concentration of 0.25 wt% was therefore chosen. IP reaction time also played a critical role in determining membrane structure and performance. Prolonging the reaction from 4 to 8 s continuously reduced AF permeance (from 652.1 to 116.1 LMH/bar) while increasing rejection (from 72.0% to 94.5%). At 10 s, no permeate was collected within 6 h, indicating that an excessively thick PA layer created very high transport resistance. These results suggest that longer IP times promote the formation of a denser selective layer, which enhances sieving capability but severely compromises water flux. Accordingly, 6 s was selected as the optimal IP time to balance permeance and rejection. In summary, both water permeance and rejection are closely related to the thickness and pore structure of the interfacial PA layer, which governs the characteristic flux-rejection trade-off.

**Figure 4:**
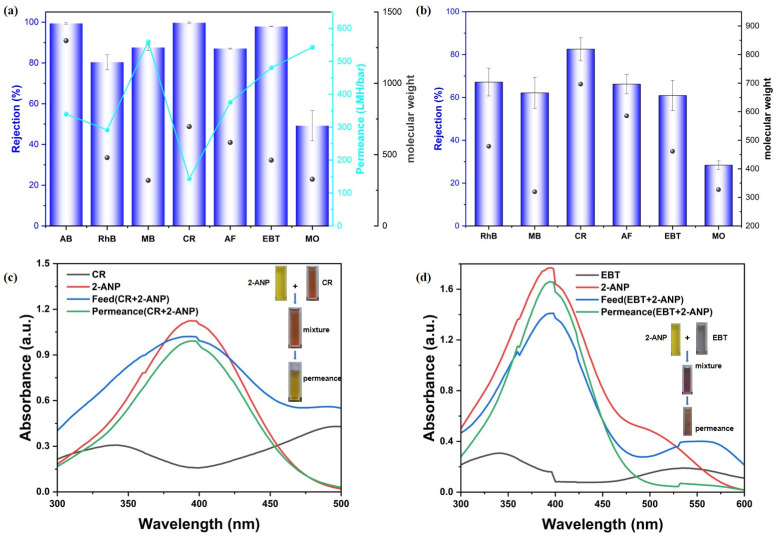
Rejection of various dyes with CM-DBC (**a**) and CM-TBDBC (**b**). Selective molecular separation of the 2-ANP-CR mixture solution (**c**) and 2-ANP-EBT mixture solution (**d**), respectively.

**Figure A2:**
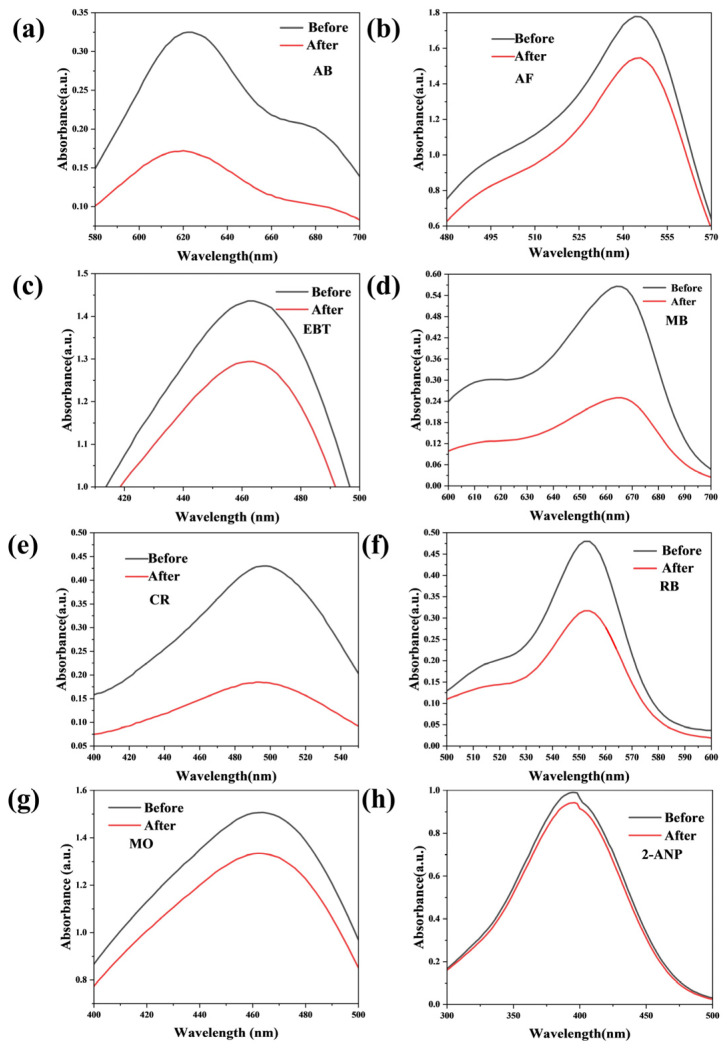
Static adsorption test of AB (**a**), AF (**b**), EBT (**c**), MB (**d**), CR (**e**), RB (**f**), MO (**g**), and 2-ANP (**h**) with the CM-DBC membrane.

Subsequently, the rejection performance of the synthesized MMMs (CM-DBC and CM-TBDBC) was assessed with various organic dyes as model contaminants. Aqueous feed solutions containing each dye at a concentration of 50 μmol/L were prepared, covering a range of molecular sizes and charges, including anionic species such as CR, (696.66 Da; ~2.56 × 0.73 nm), EBT (461.38 Da; ~1.55 × 0.88 nm), MO (327.33 Da; ~1.26 × 1.04 nm), as well as AF (585.54 Da; ~1.26 × 1.04 nm). Cationic dyes included AB (1298.86 Da; ~2.2 × 1.8 nm), RhB (479.01 Da; ~1.59 × 0.56 nm), and MB (319.85 Da; ~1.52 × 0.75 nm). Dye rejection and permeance were measured under constant pressure to evaluate membrane selectivity and permeability. Notably, dye adsorption experiments were conducted to evaluate its contribution to the overall separation performance. The results indicated that the CM-DBC membrane exhibited only minimal dye adsorption (Figure A2). As shown in Figure 4a, CM-DBC exhibited a clear selectivity between anionic dyes of comparable molecular dimensions. The rejection rates followed the order: CR (99.7%) > EBT (97.9%) > AF (87.1%) > MO (49.2%). In contrast, the cationic dyes RhB and MB showed lower retention, with rejection rates of 80.3% and 87.5%, respectively. Notably, despite its cationic nature, the large-molecular-weight dye AB was retained at 99.4%, indicating that size exclusion dominated over electrostatic interactions in this case. As to the similarly sized MB and MO, the distinct rejection behaviors are likely governed primarily by electrostatic effects, rather than by size-based sieving alone. The membrane surface is enriched with oxygen-containing and amide functional groups, which contribute to a high electron density. This characteristic appears to effectively repel the anionic MO dye, thereby leading to its lower retention. In contrast, electrostatic attraction and possible adsorption effects may partly explain the moderately higher retention observed for cationic MB. Overall, the observed trend of increasing rejection with larger molecular size further supports the contribution of a significant size-exclusion effect to the overall separation performance. However, the corresponding water permeance did not exhibit a clear trend, which may be attributed to variations in the hydrated size and aggregation state of the dye molecules during the interception process. Nevertheless, for all tested dyes, the membrane maintained a robust water permeance, consistently exceeding 143.7 LMH/bar. CM-TBDBC, fabricated under identical conditions, exhibited markedly lower rejection across all tested dyes (Figure 4b). The measured rejection rates for RhB, MB, CR, AF, EBT, and MO were 67.2%, 62.1%, 82.6%, 66.2%, 60.9%, and 28.4%, respectively. The disparity in separation performance between the two MMMs can likely be attributed to intrinsic structural differences originating from their respective COP fillers (COP-DBC vs. COP-TBDBC) and the subsequent membrane formation process. Key factors include variations in the inherent pore size and pore size distribution of the two COPs, as well as differences in the final membrane morphology (such as the shape, connectivity, and size distribution of the pores) that arise from variations in particle–polymer interaction and processing conditions during membrane fabrication. These structural distinctions ultimately govern the sieving and transport properties of each composite membrane. To further elucidate the role of the COP interlayer, control membranes were fabricated using non-functionalized COP interlayers for comparison. Under identical filtration conditions, the PA-modified membrane without any COP interlayer (NL + PA) exhibited a CR rejection of 93.0% and an AF rejection of 79.6%, both of which were lower than the corresponding values achieved by the CM-DBC membrane. Combined with the distinct dye rejection performance observed between CM-DBC and CM-TBDBC, these results demonstrate that the COP interlayer is crucial for achieving high separation performance. To further evaluate the membrane’s separation capability, selective separation studies were conducted using mixtures of organic pollutants. As a widely used key intermediate in the synthesis of acid dyes, disperse dyes, and organic pigments, 2-amino-5-nitrophenol (2-ANP) was selected as the comparative permeating molecule to evaluate the membrane’s separation selectivity. Filtration tests using mixed organic pollutants revealed that the membrane exhibited excellent selectivity: it effectively retained Congo red (CR) and Eriochrome Black T (EBT) while allowing 2-ANP to permeate freely, corresponding to a retention rate of only 11.9% (Figure 4c,d).

### 3.3. Rejection Performance Toward the Inorganic Salt and the Long-Term Stability Studies

**Figure 5:**
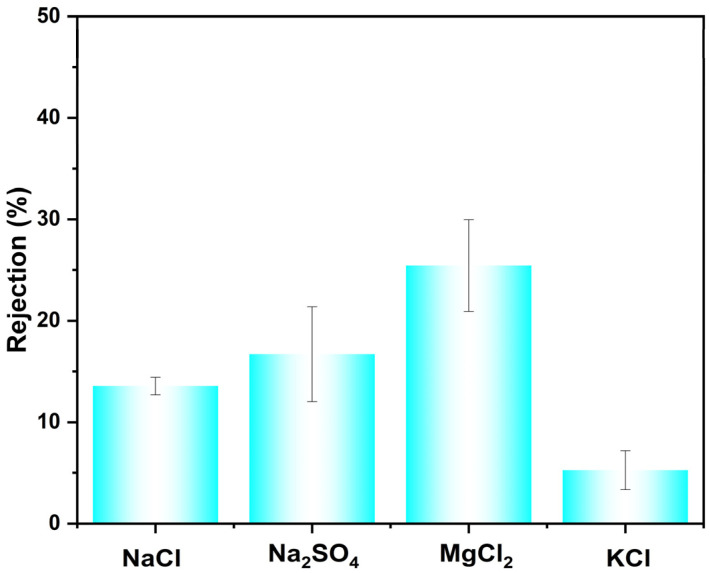
Rejection of different salts with CM-DBC.

The membrane’s rejection performance toward inorganic salts was further evaluated (Figure 5). The measured rejection rates for NaCl, Na_2_SO_4_, MgCl_2_, and KCl were 13.5%, 16.7%, 25.4%, and 5.3%, respectively. To evaluate its performance in more complex, realistic feeds, mixed-dye/salt separation experiments were conducted. Specifically, the separation performance of CR or AF from two common inorganic salts (NaCl and KCl) was experimentally tested, with the results shown in Table 3. In addition, the accuracy of the salt rejection values was ensured through a multi-step protocol: UV-Vis confirmed negligible dye in the permeate (≤2 ppm), conductivity calibration verified linear instrument response and absence of dye interference, and direct conductivity comparison showed the dye contribution (<3 μS/cm) to be insignificant relative to the salt signal (>1500 μS/cm) in the permeate. Among the tested systems, the membrane exhibited the highest separation performance for the CR/KCl mixture, achieving a selectivity of 186.8 and a flux of 138.2 LMH/bar. Selectivities for the CR/NaCl, AF/KCl, and AF/NaCl pairs were 128.5, 5.72, and 3.93, respectively. The comparatively lower selectivity observed for NaCl may be attributed to two primary factors. First, the hydrated ionic diameter of Na^+^ (0.716 nm) is larger than that of K^+^ (0.660 nm), leading to a higher retention of NaCl based on the size-exclusion effect. Second, the presence of salt in the feed solution influences the membrane’s retention of the dye molecules. The combination of these effects results in higher salt selectivity for KCl, making the membrane particularly suitable for the preliminary treatment of saline wastewater, where it can efficiently concentrate contaminants while allowing smaller salts to permeate.

**Figure 6:**
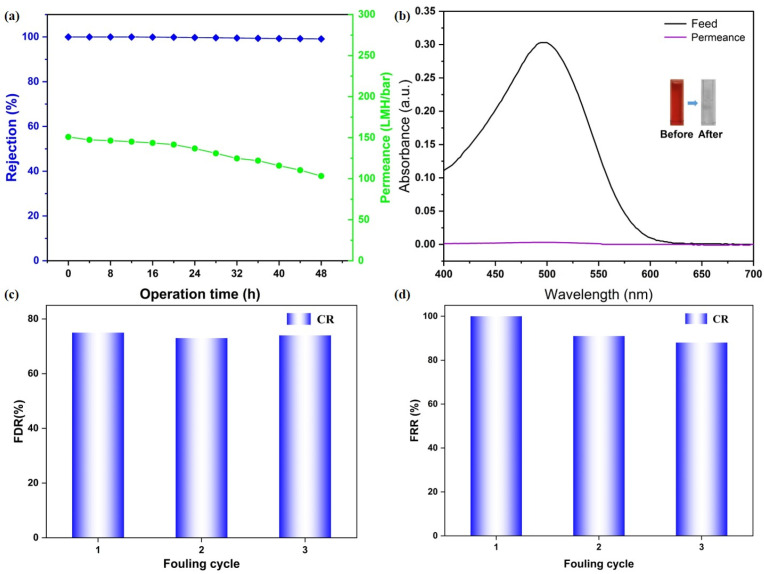
Long-term stability studies of CM-DBC (**a**) and the corresponding photos and UV–vis spectra of feed and permeate solutions (**b**). Flux decline ratio (**c**) and flux recovery ratio (**d**) analysis of CM-DBC toward CR rejection.

To evaluate the long-term separation stability of the CM-DBC membrane, a continuous filtration experiment was conducted over 48 h using a 50 ppm CR aqueous solution as the feed. As shown in Figure 6a, the membrane displayed an initial CR rejection of 99.99%, which remained essentially constant at 99.12% after the 48 h test, indicating robust retention performance over extended operation. Over the same period, water permeance gradually declined from 150.81 LMH/bar to 103.21 LMH/bar, a decrease that can be ascribed to progressive dye adsorption on the membrane surface and enhanced concentration polarization. Despite this reduction, the final permeance was still maintained at a practically useful level. Complementary UV-Vis analysis of the feed and permeate after 48 h (Figure 6b) confirmed the membrane’s sustained separation efficiency, with the characteristic CR absorption peak in the permeate being markedly lower than in the feed. What’s more, a preliminary assessment of membrane fouling resistance was performed via an antifouling test. The membrane’s fouling resistance was preliminarily assessed using cyclic filtration-cleaning tests. The FRR remained high with only slight attenuation over three cycles, indicating effective cleaning and reversible fouling behavior. Meanwhile, a comparably elevated FDR during filtration further suggests that fouling was primarily caused by loosely bound, removable deposits. Together, these metrics demonstrate that the membrane possesses promising antifouling performance and operational stability under the tested conditions. These findings collectively demonstrate that the CM-DBC membrane combines good long-term rejection stability toward CR with well-preserved water flux, underscoring its operational durability and potential for real-world implementation in advanced wastewater treatment processes.

Moreover, to assess the dye/salt separation selectivity of the membrane developed in this work, its performance was compared with other membranes reported in the literature (Table 4). The CM-DBC membrane demonstrated an average water permeance of 138.2 LMH/bar, a CR rejection of 99.5%, a KCl rejection of 6.6%, and a resulting selectivity of 186.8. Notably, the CR/KCl selectivity exhibited is favorable when compared with several representative systems, including certain COF-based nanofiltration membranes, cellulose-nanofiber-based membranes, and Prussian blue-based mixed-matrix membranes. This performance is primarily attributed to the dual functionality of the COP interlayer, which simultaneously enhances size-based sieving and provides specific interactions for dye retention, while permitting efficient salt passage.

## 4. Conclusions

In conclusion, this study successfully developed a crown ether-based COP composite membrane using a strategy combining ultrasonic dispersion and in situ interfacial polymerization. A key innovation was the construction of a tailored, mesoporous COP interlayer derived from benzocrown ethers. This interlayer was precisely engineered on a nylon support, serving as an ideal platform for the subsequent formation of a dense PA selective layer, resulting in an engineered sandwich-like thin-film composite architecture. This design significantly enhanced dye rejection, including for complex mixtures, characterized by high dye rejection (>99% for Congo red) coupled with sustained good water flux (103.2 LMH/bar). Furthermore, the membrane exhibited outstanding selectivity, achieving a dye/salt separation factor of 186.8, and demonstrated robust long-term operational stability under continuous testing conditions. By systematically elucidating the impact of interlayer properties on performance, this work provides an innovative membrane design strategy that expands the application of COP materials, contributing to the development of highly efficient and economically viable nanofiltration membranes. These findings demonstrate that the strategic design of a crown ether-based COP interlayer is a promising avenue for developing efficient membranes for wastewater remediation and resource recovery.

## Figures and Tables

**Table 1:** Influence of COP loading in MMMs on the CR and AF rejection performance.

COP-DBC Dosage (mg) *	P (LMH/bar) **	CR Rejection (%)	P (LMH/bar) ***	AF Rejection (%)
0.5	254.2	97.6	580.3	58.2
1.0	184.2	98.8	547.1	63.9
1.5	154.7	99.5	536.0	71.8
2.0	143.7	99.9	375.8	86.9
2.5	132.6	99.9	193.4	89.3

* The COP-DBC was deposited on the nylon membrane with an area of 12.56 cm^2^ through vacuum filtration. ** The water flux during CR rejection. *** The water flux during AF rejection.

**Table 2:** Influence of the PA synthesis conditions on the AF rejection performance.

Variate		P (LMH/bar)	AF Rejection (%)
Concentration of MPD (wt%)	2.0	127.1	98.4
	3.5	386.8	86.6
	5.0	690.8	64.2
Concentration of TMC (wt%)	0.15	138.2	95.5
	0.25	381.3	87.3
	0.35	165.8	92.3
Time of IP (s)	4	652.1	72.0
	6	364.7	87.9
	8	116.1	94.5
	10	-- *	-- *

* The data are unavailable.

**Table 3:** Mixed-dye/salt separation experiments.

Mixture	P (LMH/bar)	Selectivity (α)
CR/KCl	138.2	186.8
CR/NaCl	116.1	128.5
AF/KCl	337.1	5.72
AF/NaCl	290.1	3.93

**Table 4:** The performance comparison with other membranes in the literature.

Membranes	Water Permeance (LMH/bar)	CR Rejection (%)	KCl Rejection (%)	α	References
Tubular COF-LZU1	30.0	98.6	3.0	69.3	[35]
CE18/DSDA-TMC	132.0	99.3	1.4	140.9	[36]
PEI-mica/CNFs	62.2	98.9	7.1	84.5	[37]
PEI/CMCNa/PP NF	14.2	99.4	46.8	88.7	[38]
TpPa-Py	174.0	99.0	3.8	96.2	[39]
PB/TA/PPM	80.8–135.4	98.8	5.1	79.1	[40]
COF-LZU1/PAN	70.0–50.0	96.7	6.5	28.3	[41]
CM-DBC *	138.2	99.5	6.6	186.8	This work

* The data were obtained from the mixed-dye/salt separation.

## Data Availability

The original contributions presented in the study are included in the article; further inquiries can be directed to the corresponding author.

## Abbreviations

The following abbreviations are used in this manuscript:

COP	Covalent organic polymer
PA	Polyamide
IP	Interfacial polymerization
NF	Nanofiltration
TFC	Thin-film composite
LMH/bar	L m^−2^ h^−1^ bar^−1^
SPES	Sulfonated polyethersulfone
DCM	Dichloromethane
SEM	Scanning electron microscopy
MMMs	Mixed-matrix membranes
FT-IR	Fourier transform infrared
TGA	Thermogravimetric analysis
PXRD	Powder X-ray diffraction
BET	Brunauer–Emmett–Teller
BJH	Barrett–Joyner–Halenda
NLDFT	Nonlocal density functional theory
DBC	Dibenzo-18-crown-6-ether
TMC	Trimesoyl chloride
MPD	m-Phenylenediamine
CR	Congo red
AF	Acid fuchsin
RhB	RhodamineB
MB	Methylene blue
EBT	Eriochrome BlackT
MO	Methyl orange
AB	Alcian blue 8GX
CM-DBC·	Composite membrane based on COP-DBC
CM-TBDBC	Composite membrane based on COP-TBDBC
2-ANP	2-Amino-5-nitrophenol
FRR	Flux recovery ratio
FDR	Flux decline ratio
